# Supplementary material for: Identification of Acinetobacter baumannii loci for capsular polysaccharide (KL) and lipooligosaccharide outer core (OCL) synthesis in genome assemblies using curated reference databases compatible with Kaptive
Source: Microb Genom. 2020 Mar 2;6(3):e000339. doi: 10.1099/mgen.0.000339 (PMC7200062; doi:10.1099/mgen.0.000339)
Supplement: Supplementary material 1 [file mgen-6-339-s001.pdf]

Table S1. GenBank and WGS accession numbers for the sequences used to construct the *A. baumannii* KL database

K locus	Reference isolate	GenBank or WGS accession number	Base range	Reference
KL1	A1	CP010781.1	87963..110544	(1, 2)
KL2	A74	KJ459911.1	3057..27820	(1, 3, 4)
KL3	D86	KF793926.1	3248..25749	(1)
KL4	D78	JN409449.3	3247..31285	(1, 2, 5)
KL5	SDF	BK010760 [2]	1..25029	(1, 6, 7, 8, 9)
KL6	F4	KF130871.1	3248..26268	(1, 4, 10)
KL7	SGH0701	KX011025.2	3058..28708	(1, 6, 8)
KL8	BAL097	KX712116.2	724..32274	(1, 6, 11)
KL9	RUH134	JN247441.4	3250..28148	(1)
KL10	BAL_030	KY434633.1	3058..28938	(1, 12)
KL11	J9	KF002790.2	745..26607	(13)
KL12	D36	JN107991.2	3247..38792	(2, 6, 14)
KL13	Ab689	MF522810.1	724..36552	(6, 15)
KL14	D46	KF030679.2	724..21932	(16, 36)
KL15	A85	KC118540.6	9179..37114	(2, 17, 18)
KL16	D4	MF522813.1	724..23467	(19)
KL17	G7	KC118541.2	3247..25474	(2)
KL18	Ab762	MF522811.2	724..24288	(20)
KL19	RBH2	KU165787.1	724..22129	(21, 22)
KL20	A388	JQ684178.2	3250..32301	(2, 23)
KL21	G21	MG231275.1	3058..33061	(23)
KL22	LUH5537	KC526920.2 [3]	3248..27716	(24)
KL23	Ab836	MF522812.1	724..25107	(4)
KL24	RCH51	KX756650.1	724..25150	(12)
KL25	AB5075	BK008886 [2]	1..23498	(2, 25)
KL26	Ab902	MF522809.1	724..28488	(13)
KL27[1]	4190	KT266827.1	382..31000	(6, 26)
KL28	Ab908	MF522807.1	724..26378	(6)
KL29	2007-16-27-01	AMIR01000046.1 [4]	11241..36611	(13)
KL30	NIPH 190	MN166189.1	1..20785	(27)
KL31	OIFC0162	AMFH01000019.1 [4]	107741..131132	(4)
KL32	BAL 058	KT359615.1	724..22514	(3)
KL33	NIPH 67	MN166195.1	1..22775	(4, 28)
KL34	BJAB07104	CP003846.1 [4]	94048..118441	(23)
KL35	LUH5535	KC526896.2 [3]	4373..27270	(18)
KL36	Naval-72	AMFI01000021.1 [4]	38074..64065	(13)
KL37	UV1036	KX712115.1	724..21811	(24, 36)
KL38	4300STDY7045887	UFOG01000008.1 [4]	32622..55633	-
KL39	AB_2008-15-71	AMHP01000019.1 [4]	57921..78767	(21)
KL40	D141c	KP100029.1	724..24731	(2)
KL41	Naval-57	AMFP01000100 [4]	42104..65411	-
KL42	NIPH24	MN166194.1	1..22901	(4, 29)
KL43	NIPH 60	MN166192.1	1..19982	(30)
KL44[1]	NIPH 70	MN148385.1	1..26811	(6, 26)
KL45	NIPH 201	MN166190.1	1..21078	(27)
KL46	NIPH 329	MK609549.1	1..23617	(4, 9)
KL47	UV1043	KX661320.1	724..22352	(30)
KL48	NIPH 615	MN166191.1	1..20705	(27)
KL49	BAL_173	KT359616.1	724..32883	(3, 6)
KL50	OIFC047	AMFW01000039.1 [4]	35120..58660	-
KL51	WM98c	MN148384.1	1..22098	-
KL52	H32	KY434632.1	3057..23978	-
KL53	D23	MH190222.1	736..21707	(22)
KL54	RCH52	MG867726.1	724..32088	(11)

KL55	BAL 204	MN148381.1	1..24890	-
KL57	BAL_212	KY434631.1	724..26129	(31)
KL58	BAL_114	KT359617.1	724..25347	(3)
KL60	BAL_329	MN148382.1	1..24516	(12)
KL63	BAL_103	KX712117.2	724..32678	(6)
KL73	SGH0703	MF362178.1	724..36545	(15, 32)
KL74	BAL 309	MN148383.1	1..25336	-
KL77	MSHR_188	MK370019.1	1..23882	(20)
KL80	LUH3712	KC526914.2 [3]	3090..27616	(12)
KL81	LUH3713	KC526916.2 [3]	3247..29414	(10)
KL82[1]	LUH5534	KC526908.2 [3]	3248..23847	(33)
KL83	LUH5538	KC526898.2 [3]	538..26632	(13)
KL84	LUH5540	KC526902.2 [3]	3252..26477	-
KL85	LUH5543	KC526913.2 [3]	3089..27646	-
KL87[1]	LUH5547	KC526918.2 [3]	3249..32444	(13)
KL88	LUH5548	KC526910.2 [3]	3193..23919	(30)
KL89	LUH5552	KC526919.2 [3]	3159..27654	(12)
KL90	LUH5553	KC526917.2 [3]	3191..26854	(8)
KL91	1053	KM402814.1	1832..24537	(34)
KL93[1]	B11911	BK010902 [2]	1..27476	(35)
KL102	MSHR_200	MK370021.1	1..21075	(20)
KL105	625974	JEXD01000015.1 [4]	32795..58839	(13)
KL106	219_ABAU	JVPN01000008.1 [4]	31656..58916	(13)
KL107	MSHR_183	MK370022.1	1..22790	(20)
KL108	MSHR_204	MK370023.1	1..31284	(20)
KL109	MSHR_192	MK370024.1	1..24118	(20)
KL110	MSHR_203	MK370025.1	1..34861	(20)
KL111	MSHR_53	MK370026.1	1..23163	(20)
KL112	MSHR_54	MK370027.1	1..25441	(20)
KL113	MSHR_8	MK370028.1	1..29261	(20)
KL114[1]	MSHR_89	MK388214.1	1..27862	(20)
KL116	MAR-303	MK339425.1	3275..23797	(36, 37)
KL118[1]	TG00314	ASER01000021.1 [4]	37142..66310	(23)
KL119	ARLG1794	NGGP01000084.1 [4]	30036..54769	(31)
KL120	ABBL011	LLCR01000062.1 [4]	15005..38992	(9)
KL124	ABUH511	NCXX01000026.1 [4]	32588..54524	(33)
KL125	MAR13-1452	MH306195.1	5072..32981	(38)
KL128	KZ-1093	MK339428.1	3274..5469	(37)

[1] Sequence has been modified to remove IS or IS remnants for inclusion in the KL database
[2] Third Party Accession (TPA) including current nomenclature and naming
[3] Old annotations updated in this study consistent with established nomenclature scheme (1).
[4] GenBank or WGS sequence not including current nomenclature and naming

References

1. Kenyon J, Hall R. Variation in the complex carbohydrate biosynthesis loci of *Acinetobacter baumannii* genomes. PLoS One. 2013;8(4):e62160.
2. Holt K, Kenyon J, Hamidian M, Schultz M, Pickard D, Dougan G, et al. Five decades of genome evolution in the globally distributed, extensively antibiotic resistant *Acinetobacter baumannii* global clone 1. Microbial Genomics. 2016;2(2):e000052.
3. Schultz M, Thanh D, Hoan N, Wick R, Ingle D, Hawkey J, et al. Repeated local emergence of carbapenem-resistant Acinetobacter baumannii in a single hospital ward. Microbial Genomics. 2016;2(3):e000050.

4. Kenyon J, Marzaioli A, Hall R, De Castro C. Structure of the K2 capsule associated with the KL2 gene cluster of *Acinetobacter baumannii*. Glycobiology. 2014;24(6):554-63.
5. Kenyon J, Speciale I, Hall R, De Castro C. Structure of repeating unit of the capsular polysaccharide from *Acinetobacter baumannii* D78 and assignment of the K4 gene cluster. Carbohydrate Research. 2016;434:12-7.
6. Kenyon J, Marzaioli A, De Castro C, Hall R. 5,7-Di-*N*-acetylacinetaminic acid - a novel non-2-ulosonic acid found in the capsule of an *Acinetobacter baumannii* isolate. Glycobiology. 2015;25(6):644-54.
7. Arbatsky N, Kenyon J, Shashkov A, Shneider M, Popova A, Kalinchuk N, et al. The K5 capsular polysaccharide of the bacterium *Acinetobacter baumannii* SDF with the same K unit containing Leg5Ac7Ac as the K7 capsular polysaccharide but a different linkage between the K units. . Russ Chem Bull. 2019;Int. Ed. 68(1):163-7.
8. Senchenkova SN, Kenyon JJ, Jia T, Popova AV, Shneider MM, Kasimova AA, et al. The K90 capsular polysaccharide produced by *Acinetobacter baumannii* LUH5553 contains di-N-acetylpseudaminic acid and is structurally related to the K7 polysaccharide from *A. baumannii* LUH5533. Carbohydr Res. 2019;479:1-5.
9. Kenyon JJ, Arbatsky NP, Shneider MM, Popova AV, Dmitrenok AS, Kasimova AA, et al. The K46 and K5 capsular polysaccharides produced by *Acinetobacter baumannii* NIPH 329 and SDF have related structures and the side-chain non-ulosonic acids are 4-O-acetylated by phage-encoded O-acetyltransferases. PLoS One. 2019;14(6):e0218461.
10. Kenyon J, Marzaioli A, Hall R, De Castro C. Structure of the K6 capsular polysaccharide from *Acinetobacter baumannii* isolate RBH4. Carbohydrate Research. 2015;409:30-5.
11. Arbatsky N, Kenyon J, Kasimova A, Shashkov A, Shneider M, Popova A, et al. K units of the K8 and K54 capsular polysaccharides produced by *Acinetobacter baumannii* BAL 097 and RCH52 have the same structure but contain different di-N-acyl derivatives of legionaminic acid and are linked differently. Carbohydr Res. 2019;CAR_2019_238.
12. Kenyon J, Kasimova A, Shneider M, Shashkov A, Arbatsky N, Popova A, et al. The KL24 gene cluster and a genomic island encoding a Wzy polymerase contribute genes needed for synthesis of the K24 capsular polysaccharide by the multiply antibiotic resistant *Acinetobacter baumannii* isolate RCH51. Microbiology. 2017;163:355-63.
13. Kenyon J, Shashkov A, Senchenkova S, Shneider M, Liu B, Popova A, et al. *Acinetobacter baumannii* K11 and K83 capsular polysaccharides have the same 6-deoxy-l-talose-containing pentasaccharide K units but different linkages between the K units. International Journal of Biological Macromolecules. 2017;103:648-55.
14. Kenyon J, Marzaioli A, Hall R, De Castro C. Structure of the K12 capsule containing 5,7-di-*N*-acetylacinetaminic acid from A*cinetobacter baumannii* isolate D36. Glycobiology. 2015;25(8):881-7.
15. Kenyon J, Kasimova A, Notaro A, Arbatsky N, Speciale I, Shashkov A, et al. *Acinetobacter baumannii* K13 and K73 capsular polysaccharides differ only in K-unit side branches of novel non-2-ulosonic acids: di-N-acetylated forms of either acinetaminic acid or 8-epiacinetaminic acid. Carbohydrate Research. 2017;452:149-55.
16. Kenyon J, Hall R, De Castro C. Structural determination of the K14 capsular polysaccharide from an ST25 *Acinetobacter baumannii* isolate, D46. Carbohydrate Research. 2015;417:52-6.
17. Hamidian M, Kenyon J, Holt K, Pickard D, Hall R. A conjugative plasmid carrying the carbapenem resistance gene *bla*OXA-23 in AbaR4 in an extensively resistant GC1

Acinetobacter baumannii isolate. Journal of Antimicrobial Chemotherapy. 2014;69(10):2625-8.
18. Shashkov A, Liu B, Kenyon J, Popova A, MM S, Senchenkova S, et al. Structures of the K35 and K15 capsular polysaccharides of *Acinetobacter baumannii* LUH5535 and LUH5554 containing amino and diamino uronic acids. Carbohydrate Research. 2017;448:28-34.
19. Kenyon JJ, Arbatsky NP, Sweeney EL, Shashkov AS, Shneider MM, Popova AV, et al. Production of the K16 capsular polysaccharide by *Acinetobacter baumannii* ST25 isolate D4 involves a novel glycosyltransferase encoded in the KL16 gene cluster. International Journal of Biological Macromolecules. 2019;128:101-6.
20. Meumann E, Anstey N, Currie B, Piera K, Kenyon J, Hall R, et al. Genomic epidemiology of severe community-onset *Acinetobacter baumannii* infection. Microbial Genomics. 2019;5.
21. Kenyon J, Shneider M, Senchenkova S, Shashkov A, Siniagina M, Malanin S, et al. The K19 capsular polysaccharide of *Acinetobacter baumannii* is produced via a Wzy polymerase encoded in a small genomic island rather than the KL19 capsule gene cluster. Microbiology. 2016;162:1479-89.
22. Shashkov A, Kenyon J, Arbatsky N, Shneider M, Popova A, Knirel Y, et al. Genetics of biosynthesis and structure of the K53 capsular polysaccharide of *Acinetobacter baumannii* D23 made up of a disaccharide K unit. Microbiology. 2018;164:1289-92.
23. Kasimova A, Kenyon J, Arbatsky N, Shashkov A, Popova A, Shneider M, et al. *Acinetobacter baumannii* K20 and K21 capsular polysaccharide structures establish roles for UDP-glucose dehydrogenase Ugd2, pyruvyl transferase Ptr2 and two glycosyltransferases. Glycobiology. 2018;28(11):876-84.
24. Arbatsky N, Shneider M, Kenyon J, Shashkov A, Popova A, Miroshinikov K, et al. Structure of the neutral capsular polysaccharide of *Acinetobacter baumannii* NIPH146 that carries the KL37 capsule gene cluster. Carbohydrate Research. 2015;413:12-5.
25. Senchenkova S, Shashkov A, Popova A, Shneider M, Arbatsky N, Miroshinikov K, et al. Structure elucidation of the capsular polysaccharide of *Acinetobacter baumannii* AB5075 having the KL25 capsule biosynthesis locus. Carbohydrate Research. 2015;408:8-11.
26. Shashkov A, Kenyon J, Senchenkova S, Shneider M, Popova A, Arbatsky N, et al. *Acinetobacter baumannii* K27 and K44 capsular polysaccharides have the same K unit but different structures due to the presence of distinct *wzy* genes in otherwise closely related K gene clusters. Glycobiology. 2016;26(5):501-8.
27. Shashkov A, Kenyon J, Arbatsky N, Shneider M, Popova A, Miroshinikov K, et al. Structures of three different neutral polysaccharide of Acinetobacter baumannii, NIPH190, NIPH201, and NIPH615, assigned to K30, K45, and K48 capsule types, respectively, based on capsule biosynthesis gene clusters. Carbohydrate Research. 2015;417:81-8.
28. Arbatsky N, Shneider M, Shashkov A, Popova A, Miroshnikov K, Volozhantsev N, et al. Structure of the *N*-acetylpseudaminic acid-containing capsular polysaccharide of *Acinetobacter baumannii* NIPH67. Russian Chemical Bulletin. 2016;65(2):588–91.
29. Senchenkova S, Popova A, Shashkov A, Shneider M, Mei Z, Arbatsky N, et al. Structure of a new pseudaminic acid-containing capsular polysaccharide of *Acinetobacter baumannii* LUH5550 having the KL42 capsule biosynthesis locus. Carbohydrate Research. 2015;407:154-7.
30. Shashkov A, Kenyon J, Arbatsky N, Shneider M, Popova A, Miroshnikov K, et al. Related structures of neutral capsular polysaccharides of *Acinetobacter baumannii*

isolates that carry related capsule gene clusters KL43, KL47, and KL88. Carbohydrate Research. 2016;435:173-9.
31. Kenyon J, Kasimova A, Shashkov A, Hall R, Knirel Y. *Acinetobacter baumannii* isolate BAL_212 from Vietnam produces the K57 capsular polysaccharide containing a rarely occurring amino sugar N-acetylviosamine. Microbiology. 2018;164:217-20.
32. Kenyon J, Notaro A, Hsu LY, De Castro C, Hall R. 5,7-Di-N-acetyl-8-epiacinetaminic acid: A new non-2-ulosonic acid found in the K73 capsule produced by an *Acinetobacter baumannii* isolate from Singapore. Scientific Reports. 2017;7:11357.
33. Kasimova A, Kenyon J, Arbatsky N, Shashkov A, Popova A, Knirel Y, et al. Structure of the K82 capsular polysaccharide from *Acinetobacter baumannii* LUH5534 containing a D-galactose 4,6-pyruvic acid acetal Biochemistry (Moscow). 2018;83(7):831-5.
34. Shashkov A, Shneider M, Senchenkova S, Popova A, Nikitina A, Babenko V, et al. Structure of the capsular polysaccharide of *Acinetobacter baumannii* 1053 having the KL91 capsule biosynthesis gene locus. Carbohydrate Research. 2015;404:79-82.
35. Kasimova A, Shneider M, Arbatsky N, Popova A, Shashkov A, Miroshnikov K, et al. Structure and gene cluster of the K93 capsular polysaccharide of *Acinetobacter baumannii* B11911 containing 5-N-Acetyl-7-N-[(R)-3-hydroxybutanoyl]pseudaminic acid. Biochemistry (Moscow). 2017;82(4):483-9.
36. Shashkov A, Cahill S, Arbatsky N, Westacott A, Kasimova A, Shneider M, et al. *Acinetobacter baumannii* K116 capsular polysaccharide structure is a hybrid of the K14 and revised K37 structures. Carbohydr Res. 2019;CAR_2019_291.
37. Arbatsky N, Kasimova A, Shashkov A, Shneider M, Popova A, Shagin D, et al. Structure of the K128 capsular polysaccharide produced by Acinetobacter baumannii KZ-1093 from Kazakhstan. Carbohydr Res. 2019.
38. Arbatsky N, Shneider M, Dmitrenok A, Popova A, Shagin D, Shelenkov A, et al. Structure and gene cluster of the K125 capsular polysaccharide from *Acinetobacter baumannii* MAR13-1452. International Journal of Biological Macromolecules. 2018;117:1195-9.

Table S2. GenBank and WGS accession numbers for the sequences used to construct the *A. baumannii* OCL database

OC locus	Reference isolate	GenBank or WGS accession number	Base range	Reference
OCL1	A1	CP010781.1	3366405..3375181	(1-3)
OCL2	D36	CP012952.1	646907..655270	(1, 3)
OCL3	A85	CP021782.1	3464825..3473737	(1, 3)
OCL4	A388	CP024418.1	642040..650673	(3)
OCL5	G21	MG231275.1	37120..46166	(3)
OCL6	D46	KF030679.1	28675..37977	(3)
OCL7	OIFC035	AMTB01000038.1 [1]	221522..230586	(3)
OCL8	OIFC111	AMFY01000013.1 [1]	222496..228777	(3)
OCL9	Naval-72	AMFI01000027.1 [1]	34336..40843	(3)
OCL10	AB_TG27343	AMIS01000032.1 [1]	97841..111169	(3)
OCL11	TG22204	ASFV01000009.1 [1]	33588..43841	(3)
OCL12	NIPH 410	ATGJ01000006.1 [1]	47175..57655	(3)

[1] GenBank or WGS sequence not including current nomenclature and naming

References

1. Kenyon J, Hall R. Variation in the complex carbohydrate biosynthesis loci of *Acinetobacter baumannii* genomes. PLoS One. 2013;8(4):e62160.
2. Kenyon J, Holt K, Pickard D, Dougan G, Hall R. Insertions in the OCL1 locus of *Acinetobacter baumannii* lead to shortened lipooligosaccharides. Research in Microbiology. 2014;165(6):472-5.
3. Kenyon J, Nigro S, Hall R. Variation in the OC locus of *Acinetobacter baumannii* genomes predicts extensive structural diversity in the lipoligosaccharide. PLoS One. 2014;9(9):e107833.
